# Tai Chi and vestibular rehabilitation improve vestibulopathic gait via different neuromuscular mechanisms: Preliminary report

**DOI:** 10.1186/1471-2377-5-3

**Published:** 2005-02-18

**Authors:** Chris A McGibbon, David E Krebs, Stephen W Parker, Donna M Scarborough, Peter M Wayne, Steven L Wolf

**Affiliations:** 1 Institute of Biomedical Engineering, University of New Brunswick, Fredericton, NB E3B5A3, Canada; 2 Biomotion Laboratory, Massachusetts General Hospital, Boston, MA 02114, USA; 3 MGH Institute of Health Professions, Boston, MA 02129, USA; 4 Harvard Medical School, Boston, MA 02115, USA; 5 Dept of Neurology, Massachusetts General Hospital, Boston, MA 02114, USA; 6 New England School of Acupuncture, Watertown, MA, 02472, USA; 7 Dept of Rehabilitation Medicine, Emory University School of Medicine, Atlanta, GA 30322, USA

## Abstract

**Background:**

Vestibular rehabilitation (VR) is a well-accepted exercise program intended to remedy balance impairment caused by damage to the peripheral vestibular system. Alternative therapies, such as Tai Chi (TC), have recently gained popularity as a treatment for balance impairment. Although VR and TC can benefit people with vestibulopathy, the degree to which gait improvements may be related to neuromuscular adaptations of the lower extremities for the two different therapies are unknown.

**Methods:**

We examined the relationship between lower extremity neuromuscular function and trunk control in 36 older adults with vestibulopathy, randomized to 10 weeks of either VR or TC exercise. Time-distance measures (gait speed, step length, stance duration and step width), lower extremity sagittal plane mechanical energy expenditures (MEE), and trunk sagittal and frontal plane kinematics (peak and range of linear and angular velocity), were measured.

**Results:**

Although gait time-distance measures were improved in both groups following treatment, no significant between-groups differences were observed for the MEE and trunk kinematic measures. Significant within groups changes, however, were observed. The TC group significantly increased ankle MEE contribution and decreased hip MEE contribution to total leg MEE, while no significant changes were found within the VR group. The TC group exhibited a positive relationship between change in leg MEE and change in trunk velocity peak and range, while the VR group exhibited a negative relationship.

**Conclusion:**

Gait function improved in both groups consistent with expectations of the interventions. Differences in each group's response to therapy appear to suggest that improved gait function may be due to different neuromuscular adaptations resulting from the different interventions. The TC group's improvements were associated with reorganized lower extremity neuromuscular patterns, which appear to promote a faster gait and reduced excessive hip compensation. The VR group's improvements, however, were not the result of lower extremity neuromuscular pattern changes. Lower-extremity MEE increases corresponded to attenuated forward trunk linear and angular movement in the VR group, suggesting better control of upper body motion to minimize loss of balance. These data support a growing body of evidence that Tai Chi may be a valuable complementary treatment for vestibular disorders.

## Background

Vestibulopathy decreases whole body dynamic postural control and causes functional limitations [1-4]. Limitations in ambulation, dynamic balance and trunk control, for example, can lead to disability and contribute to decreased quality of life [5]. Vestibular rehabilitation (VR) is a well-accepted exercise program intended to remedy balance impairment caused by damage to the peripheral vestibular system [6]. Vestibulopathy impairs both the vestibulo-ocular reflex (VOR) and the vestibulo-spinal reflexes (VSR) [7]; hence, VR is designed to adapt the CNS to diminished vestibular input and to compensate for VOR and VSR loss, via gaze and balance retraining, which in turn should improve whole body dynamic stability [8-10].

Alternative therapies, such as Tai Chi (TC), have recently gained popularity as a treatment paradigm for a variety of human ailments, including balance impairment [11-13]. TC employs detailed regimens of physical movement, breathing techniques, and cognitive tools to strengthen the body, relax the mind, and balance the flow of life force [14]. The purported improvements in overall body control and mind-body focus with TC may offer an improved approach to treating balance dysfunction [11,13,15-17]. Where the explicit objective of many VR exercises is to improve gaze stability, TC emphasizes a 'soft' unfocussed gaze during the prescribed balance exercises. Although there is strong evidence that VR [4,8-10,18], and more recently TC [11,13,15-17], can benefit people with vestibulopathy, the degree to which gait improvements may be related to neuromuscular adaptations of the lower extremities are unknown. Thus, although the end result of both TC and VR should be improved dynamic stability during locomotor activities of daily living, including gait, we hypothesized that the *mechanisms *underlying such improvements should differ substantially. A better understanding of how balance dysfunction interventions affect lower extremity neuromuscular function during ADL may be useful for developing gait training exercises, and for providing a fuller understanding of the link between motor function and balance.

In this report, we present preliminary data from a blinded randomized clinical trial comparing the effects of VR and TC on gait function, joint kinetics and trunk kinematics in older adults. While the overall aim of this study was to determine the effects of balance rehabilitation on gait characteristics, we directed our efforts in this paper to better understand the relationship between mechanical energy transfers along the lower extremity kinematic chain (ankle-knee-hip), and forward and side-to-side velocity of the trunk.

Our general hypothesis was that adults with balance impairment from vestibulopathy who receive the VR or TC intervention will improve gait function as indicated by time-distance measures. Our specific hypotheses are based on the following rationale: Recent studies in healthy older adults [19-21], with general impairments such as strength loss [22], or pathologies such as knee arthritis [23,24], show that the hip musculature often aids, or compensates for, ankle plantar-flexor muscles in providing both forward propulsion and trunk stability [25]. These prior studies have shown a consistent decline in plantar-flexor muscle power during gait, with an increase in hip muscle power, in older adults with, and without, known mobility impairments. As shown recently by Neptune and colleagues [26], the ankle plantar flexors contribute significantly to both forward propulsion and vertical trunk stability. Thus, one would expect that improvements in lower extremity motor control, aimed at increasing forward propulsion and trunk stabilization, would be represented by decreases in hip mechanical energy expenditures and increases in ankle (and perhaps knee) mechanical energy expenditures, and be directly related to improved kinematics of the trunk.

Based on the above rationale, and the specific treatment programs described in the following sections, we hypothesized: 1) TC treatment will improve lower extremity motor control by increasing ankle mechanical energy expenditure (MEE) contribution, and decreasing hip MEE contributions, to total energy of the leg, more than VR; and 2) that improved trunk control following TC will be positively correlated with improvements in lower extremity motor control, while improvements in trunk control following VR will not be correlated with improvements in lower extremity motor control. The latter may indirectly implicate other mechanisms, most likely improvements in VOR/gaze stability [27].

## Methods

### Subjects

Fifty-three patients with balance impairment due to vestibular hypofunction were recruited and randomized into two treatment groups: VR, a group vestibular rehabilitation intervention, and TC, a group Tai Chi exercise. Of the 53 patients admitted, 15 dropped out or were excluded prior to completing the intervention. The majority of drop outs were due to a new medical condition unrelated to the study preventing participation (e.g. fractured foot, acute back pain) or due to the sudden need to care for an ill family member. Two subjects were eliminated because of lack of force plate data to use in the data analysis (see Gait Analysis section for more detail). Of the 36 subjects remaining for analysis, 17 subjects were randomized into the VR treatment group (12 unilateral and 5 bilateral) and 19 subjects in the TC group (11 unilateral, 8 bilateral).

Unilateral or bilateral vestibular hypofunction (UVH or BVH) diagnoses were obtained as previously described [10]. Briefly, all patients had gait unsteadiness without evidence of central nervous dysfunction. All patients were referred to the study because they had locomotor instability for which they sought treatment from project physicians. Patients with bilateral vestibular hypofunction had bilaterally decreased caloric responses (total slow phase velocity of ≤10 degrees·sec^-1 ^for the sum of right and left ear caloric stimulation at 27 and 44°C warm water stimulation of both ears *and *≤8°·sec^-1 ^slow phase velocity for the sum of 35 cc of ice water stimulation in each ear) and decreased VOR gains during passive rotational testing at up to 50°·sec^-1 ^(at least 2.5 SD below normal mean values at frequencies of rotational testing from .01 to 0.5 Hz). Patients with unilateral vestibular hypofunction had damage only on one side, including at least 30% unilaterally reduced caloric response, positional nystagmus while lying with the damaged ear down, and/or confirmatory abnormalities on rotational testing (mildly decreased low frequency gains, increased phase leads and asymmetrical rotation induced nystagmus, i.e., decreased vestibular time constant). Patients with bilateral deficits are typically more disabled than are those with unilateral deficits. The average time post-onset of vestibulopathy for the 36 subjects included in the analysis was 3.05 years (range 0.58 – 12 years). All subjects were community dwelling and reported varying degrees of limitations in locomotor activity. Twenty of the 36 subjects were female, and the 36 subjects were 59.5 ± 11.5 years old (range, 41–81), 1.70 ± .11 m tall and 83.6 ± 16.5 kg in weight (breakdown by treatment group is shown in the Results section). All subjects had at least one course of VR since the time of onset of their vestibular symptoms. Inclusion criteria required that each subject did not have VR for >6 months from study enrollment. The testing protocol was approved by MGH institutional review board, and all subjects provided written informed consent according to institutional guidelines on human research.

### Interventions

The VR and TC treatment interventions were provided in a total of six (3 VR and 3 TC groups) small groups with an average of 8 subjects per group. Each intervention program met once weekly, on separate weekdays, for 10 weeks in the same exercise room. The weekly sessions for both intervention groups lasted approximately 70 minutes. Each treatment program was lead by the same instructor for the three treatment cohorts. The instructors were blinded to the exercises provided to the other treatment program. One or two assistants were available for each session for all treatment groups to insure participants' safety. All treatment sessions included time to: 1) review material introduced in prior sessions; 2) introduce new material; 3) ask questions and share personal experiences or concerns regarding the practices; and 4) cool down and rest.

#### Tai Chi intervention

The TC intervention incorporated three objectives outlined in a balance-related TC program developed by Wolf and colleagues [12,17]. First, it emphasized movements that are easily comprehensible. Second, the sequence of exercises introduced reflected a progression that increasingly challenges postural stability, with a shift in weight bearing from bilateral to unilateral support. Third, the program emphasized increasing the magnitude of trunk and arm rotation while diminishing the base of support. The five specific TC movements employed in this study – 'raising the power', 'withdraw and push', 'wave hand like clouds', 'brush knee twist step', and 'separate right and left legs' – are described and illustrated in a training manual for the Cheng Man-Ch'ing's Yang-style short form [28]. In addition to these five formal TC movements, the intervention also included a short set of traditional TC warm-up exercises focused on loosening up the physical body and incorporating mindfulness and imagery into movement. Warm-up exercises included: gentle stretches sequentially targeting the shoulders, necks, arms and legs; a torso stretching exercise that coordinated weight shifts with rotations of the trunk and passive arm swinging; and a 5 minute seated meditation emphasizing relaxed diaphragmatic breathing. Approximately 20 minutes of each class was devoted to warm-up exercises, of which 10–12 minutes was spent in standing. Following an additional 40 minutes of formal TC practice, 10 minutes was allowed for group discussion.

#### Vestibular rehabilitation intervention

The VR intervention used in this study was a comprehensive exercise program designed to improve the problems specifically associated with damage to the peripheral vestibular system [4,6,10,29]. Each treatment session focused on the three main objectives of the VR intervention. Firstly, a series of eye-head coordination exercises were performed to promote gaze stability during both quiet standing and dynamic functional activities (such as combining movement of an image across the retina with head movement). Subjects progressed to performing these eye-head exercises with the target on a more complex background (to simulate real world activities), at increasingly faster speeds of head movements (eg, 2–3 Hz), and during more dynamic standing and locomotor activities. A second treatment objective included VOR training in a group format with subjects standing. Target foveation objects (words of various sizes) were fixed to a large checkerboard background covering one wall of the exercise room, enabling us to provide the appropriate visual stimuli. Patients were progressed by increasing the speed (frequency and amplitude) of head movement to train the VOR more appropriately at speeds consistent with everyday locomotor activities. The third main component of the VR program was upright balance retraining exercises that enhance the use of various sensory cues for gaining posture control [8,29,30]. Examples of these exercises include subjects maintaining their balance while decreasing their base of support (such as standing on one foot, marching or walking heel to toe) and while walking on various floor surfaces (such as the pliable surfaces of a foam mat). Subjects were further challenged by incorporating head and trunk movements or with eyes closed during standing and walking exercises. All exercises were performed in an upright position (either standing or during locomotion) based on individual tolerance. If required, a seat was provided and the exercise performed in a seated position until the subject was able to tolerate the activity in standing. Each group treatment session lasted 60 minutes allowing 20 minutes for each of the 3 main exercise components. There was an additional 10 minutes for questions and answer time and for assistance with individual progression of home exercise programs.

### Gait analysis

Subjects performed two-to-four gait trials along a 10 m level walkway at baseline testing, and at testing following the intervention program, at their freely selected pace upon the command "Please walk as you normally would, as if taking a brisk walk in the park". Body segment kinematics were acquired at 150 Hz with a four-camera Selspot optoelectric tracking system (Selective Electronics, Partille, Sweden), used to acquire position and orientation data of 11 segments (both feet, shanks, thighs and arms, and the pelvis, trunk and head). Collection of kinematic data and processing is described in more detail elsewhere [31]. Kinetic data consisted of ground reaction forces acquired from two adjacent piezoelectric force platforms (Kistler Instruments, Winterthur, Switzerland), synchronously sampled with body segment kinematic data at 150 Hz. Subjects were required to have at least one good gait trial, both at baseline and post-intervention testing sessions, to be included in the data analysis. A "good gait trial" was one that satisfied the following criteria: 1) one foot was required to be in whole contact with only one or both force platforms without interference from the other foot, 2) all body segments were visible, and tracked without artifact, during the stance portion of gait. Two subjects were excluded on the basis of failing one or both of the above criteria.

### Data analysis

Parameters selected for data analysis consisted of dynamic gait function (time-distance measures), lower extremity neuromuscular control (sagittal plane mechanical energy expenditures, MEE), and trunk stability (sagittal and frontal plane kinematics).

#### Dynamic gait function

Gait function was assessed with standard time-distance measures [4,32], including: gait speed, step length, step width and stance duration. Gait speed was measured as the average anterior-posterior velocity component of the whole-body center of gravity over stance phase of gait. Step length was measured as the anterior-posterior distance between right and left ankle centers when each foot was flat on the floor during its respective mid stance portion. Stance duration was measured as the time elapsed between heel strike and toe off (duration of stance phase), and step width was measured as the lateral distance between ankle centers at the foot positions used for step length calculation.

#### Lower extremity neuromuscular function

Neuromuscular function of the lower extremities was assessed using mechanical energy expenditure (MEE) of the ankle, knee and hip, relative to the total MEE of the leg, and were computed as described previously [23,33]. Briefly, the mechanical power profile of the joint, the scalar product of net joint moment and angular velocity, is integrated over specific time intervals to arrive at mechanical energy expended, MEE, or work done. The intervals are defined by periods of concentric transfer (MEE^(+)^, the amount of concentric mechanical energy expended with segment-to-segment energy transfer), eccentric transfer (MEE^(-)^, the amount of eccentric mechanical energy expended with segment-to-segment energy transfer), and no-transfer (MEE^(o)^, the amount of concentric and eccentric energy expended without segment-to-segment energy transfer) conditions. The total joint MEE is simply the sum of these components (MEE^(t) ^= MEE^(+) ^+ MEE^(-) ^+ MEE^(o)^). Leg MEE is the sum of joint MEE (ankle, knee and hip) for different conditions, or totals. Percentage contribution of joint MEE (for each condition and total) to leg MEE (for each condition and total) was then calculated.

#### Trunk stability

Trunk stability was assessed using kinematics of the trunk center of mass [34], and consisted of anterior-posterior trunk velocity (peak and range) as well as lateral trunk velocity (peak and range); sagittal plane angular (pitch) velocity of the trunk (peak and range) and frontal plane angular (roll) velocity of the trunk (peak and range). The rationale for using kinematic measures of trunk stability, instead of overall stability (such as whole-body CG sway or kinematics), however, was to enable us to examine the relationship between lower extremity neuromuscular function (using the mechanical energy analysis as such a measure) and the kinematics of the upper body, as a mass to be controlled apart from the legs. Peaks and ranges were taken from stance phase of the same leg used for the mechanical energy analysis described above.

### Statistical analysis

One-way ANCOVA was used to compare change scores between the two groups, using the baseline values as covariates. Paired samples t-tests compared the change in each variable for each group from baseline to post-intervention testing. Pearson correlations were used to examine associations between change scores in lower extremity MEE and change scores in trunk velocities, for each treatment group. Due to the large number of comparisons in this exploratory study, a Ryan-Holm step down Bonferonni approach was used to control for type I errors [35], using a family-wise α = .05. Using this scheme, families of three members (MEE^(+)^, MEE^(-) ^and MEE^(o) ^contributions) required significance at α = .017 for at least one comparison, α = .025 for the second comparison, and α = .050 for the third comparison. Families of four members (anterior-posterior peak and range, and lateral peak and range of trunk velocity) required significance at α = .013 for at least one comparison, α = .017 for the second comparison, and so on. All p-values given will be unadjusted, but the adjusted α is given for each comparison where appropriate. SPSS for Windows (v10, SPSS Inc. Chicago, IL) was used for all statistical analyses.

## Results

The two groups were not different in age (VR: 56.9 ± 11.6 yrs; TC: 61.7 ± 11.3 yrs; *p *= .223), height (VR: 1.69 ± .11 m; TC: 1.71 ± .11 m; *p *= .712) or weight (VR: 81.1 ± 19.3 kg; TC: 85.8 ± 13.6 kg; *p *= .399). There was no significant difference in proportion of UVH and BVH in the treatment groups (Chi-square, p = .429), or proportion of men and women in the treatment groups (Chi-square, *p *= .709).

There were no significant between-groups differences (using ANCOVA for controlling for baseline differences) for any of the variables examined. There were, however, significant changes pre- and post-treatment within each group. These latter results appear to suggest that clinically important differences in each group's response to the therapies exist. Thus, the remainder of the results presented will focus on the within-groups comparisons.

### Time distance measures

Both groups improved (unadjusted α = .05) following intervention in time-distance measures (see Table 1), with the TC group showing greater overall improvements; the VR group improved significantly in stance duration (*p *= .044) and step length (*p *= .045), but not in gait speed (*p *= .060) or step width (*p *= .390); the TC group improved in gait speed (*p *= .009) and step length (*p *= .010), but not in stance duration (*p *= .055) or step width (*p *= .313).

**Table 1:** Time distance measures before and after intervention.

	Variable	Baseline Value		Post-Treatment Value		
	
		Mean	Standard Dev	Mean	Standard Dev	*p*-value*
VR	Gait speed (m/s)	1.180	.312	1.235	.229	.060
	Step length (m)	.616	.119	.639	.116	.045
	Stance duration (s)	.667	.065	.653	.047	.044
	Step width (m)	.093	.045	.096	.040	.390

TC	Gait speed (m/s)	1.090	.275	1.170	.261	.009
	Step length (m)	.582	.110	.612	.118	.010
	Stance duration (s)	.715	.089	.684	.055	.055
	Step width (m)	.109	.046	.114	.042	.313

* Within-groups paired t-test significance, based on unadjusted α = .05.

### Mechanical energy expenditures

**Figure 1:**
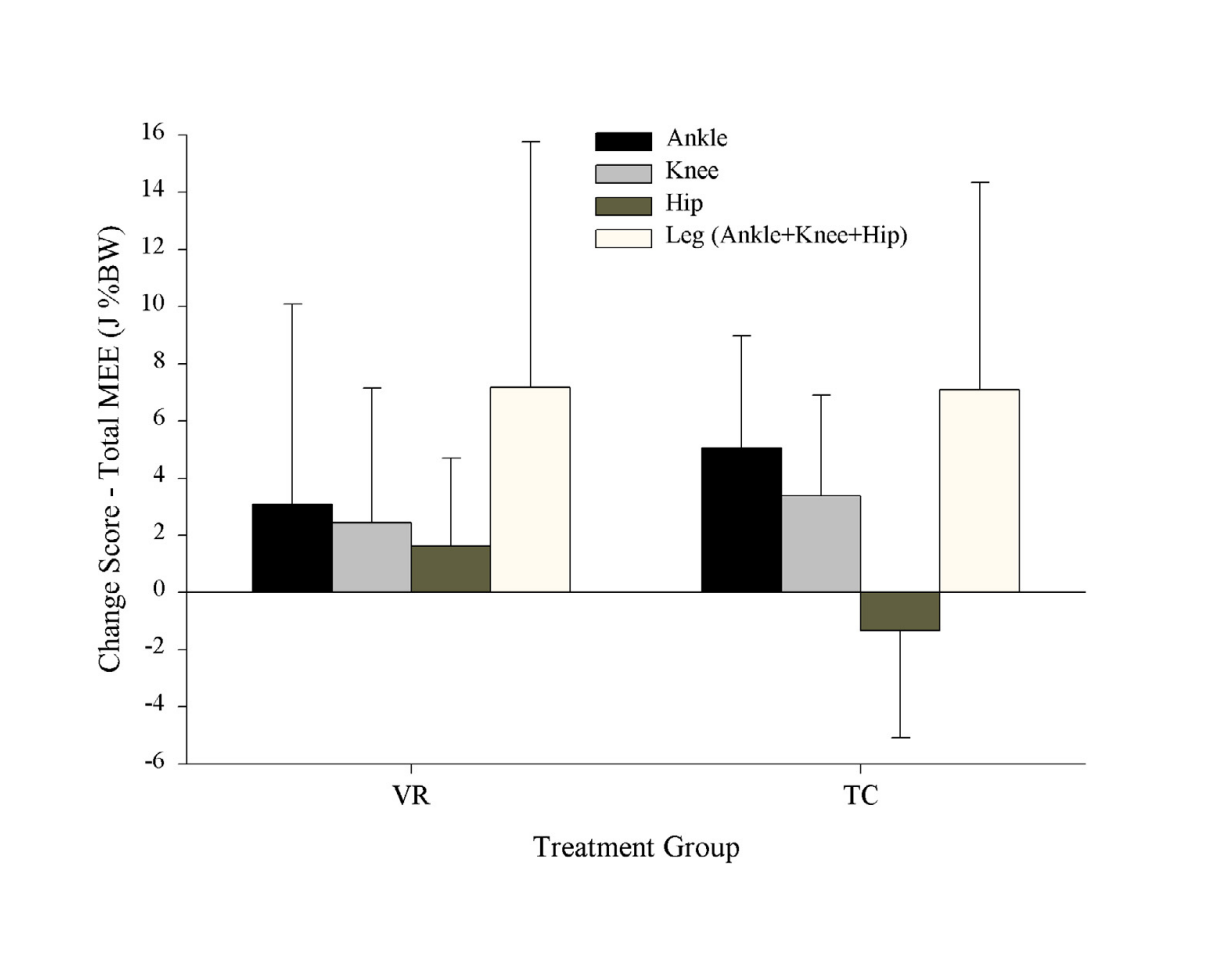
Change scores in ankle, knee and hip total MEE^(t) ^and leg total MEE^(t) ^for VR and TC groups (in J %BW). Error bars represent 95% confidence intervals on the mean.

**Figure 2:**
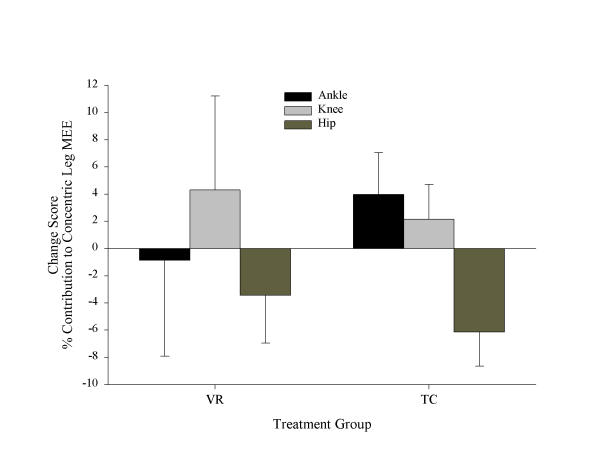
Change scores in percent contribution of ankle, knee and hip concentric MEE^(+) ^to leg concentric MEE^(+) ^for VR and TC groups (in J %BW). Error bars represent 95% confidence intervals on the mean.

Figure 1 shows the changes in joint and leg MEE^(t) ^for each joint, and the sum of all the joints (leg). Although the total leg MEE^(t) ^change was similar, the distribution of joint MEE^(t) ^were quite different for the two treatment groups. Comparison of the change in percent contribution of MEE for each transfer condition for each joint to leg MEE showed that only the TC group had significantly reduced (*p *< .001, adjusted α = .017) relative hip concentric MEE^(+) ^and increased (*p *= .019, adjusted α = .025) relative ankle concentric MEE^(+)^, following training. These data are shown in Figure 2.

### Trunk kinematics

**Figure 3:**
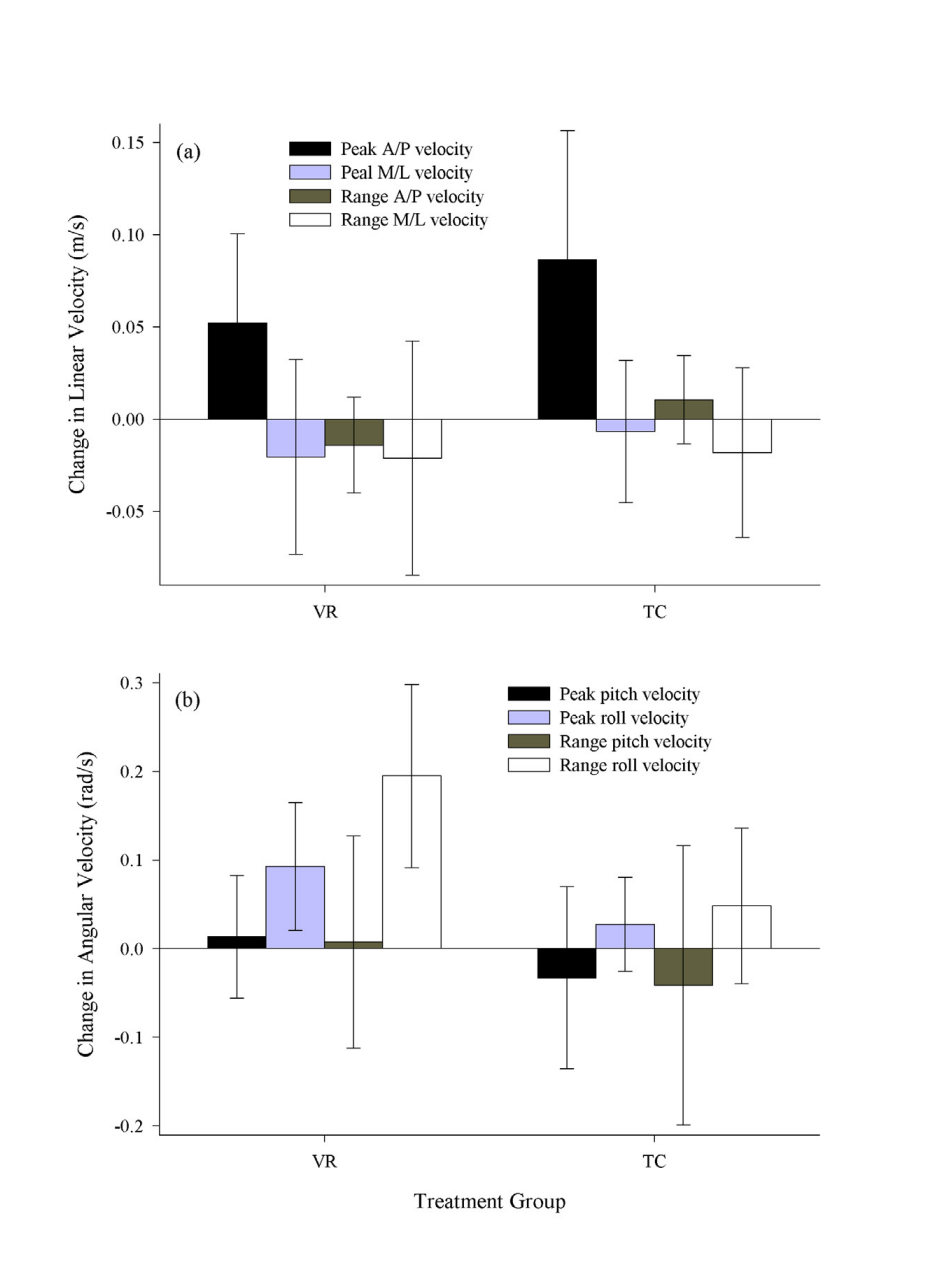
Change scores in trunk velocity. (a) Linear velocity: anterior-posterior (A/P) velocity peak and range, and medial-lateral (M/L) velocity peak and range; (b) Angular velocity: pitch (sagittal plane) velocity peak and range, roll (frontal plane) velocity peak and range. Error bars represent 95% confidence intervals on the mean.

TC group had significantly increased (*p *= .009, adjusted α = .013) peak trunk forward velocity during stance phase of gait following treatment, while the VR group's increase was similar though not statistically significant (*p *= .018, adjusted α = .013). There were no significant changes in forward velocity range, nor were there significant changes in peak or range of lateral trunk velocity for either group. The VR group, however, did show a significant increase in peak trunk angular velocity (*p *= .007, adjusted α = .017) and range of trunk angular velocity (*p *< .001, adjusted α = .013) in the frontal plane. There were no significant changes in trunk angular velocity in the frontal plane for the TC group, and neither group showed significant changes in peak and range of trunk angular velocity in the sagittal plane. These data are summarized in Figure 3.

### Relationships between MEE and trunk kinematics

**Figure 4:**
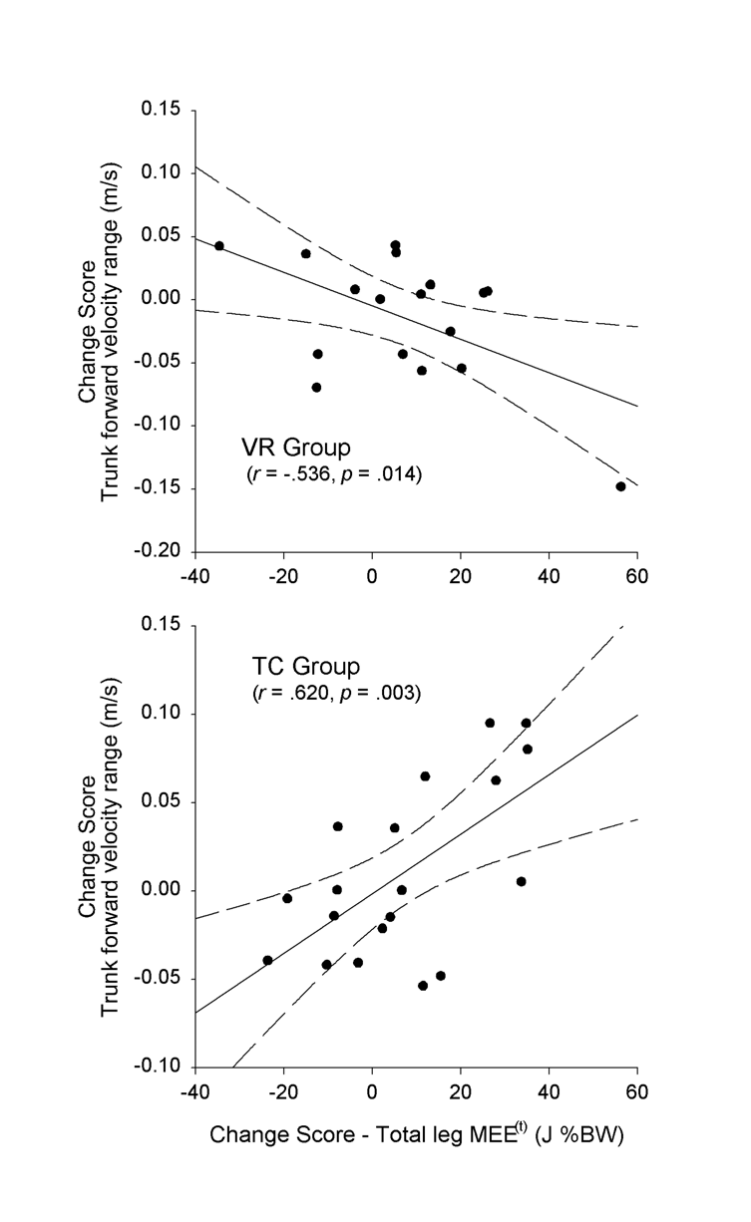
Change scores in trunk forward velocity range (in m/s) versus change scores in total leg MEE^(t) ^(in J %BW) for VR (top plot) and TC (bottom plot) groups. Dashed lines represent the 95% confidence intervals on the mean.

Correlation analysis between changes scores in leg MEE and trunk kinematics revealed significant relationships for both treatment groups. Most striking was the consistent directional relationship between trunk velocity and leg MEE within each of the treatment groups. For the VR group, changes in range and peak of forward velocity of the trunk was negatively correlated with changes leg MEE (range: *r *= -.536, *p *= .013, adjusted α = .013; peak: *r *= -.431, *p *= .042, adjusted α = .017). For the TC group, however, changes in range and peak of forward velocity of the trunk was positively correlated with changes in leg MEE (range: *r *= .620, *p *= .003, adjusted α = .013; peak: *r *= .451, *p *= .026, adjusted α = .017). Figure 4 shows scatter plots depicting the positive and negative relationships between change scores in leg MEE and trunk velocity range for TC and VR groups, respectively. There were no significant relationships detected between change in joint or leg MEE and change in lateral linear velocity of the trunk, nor in sagittal or frontal plane angular velocity of the trunk.

## Discussion and conclusions

Little is known about the mechanisms of improved balance and postural control following rehabilitation in people with vestibulopathy. Although VR has shown promise for improving patients balance and gaze stability [4,9,10,29,36-40], just over 65% of people treated respond to the therapy [10]. Improvements in function are not ubiquitous with VR treatment. Alternative therapies, such as TC, offer a complementary approach to improving balance and postural control by teaching body control and awareness [12,15]. The purpose of the present study was to examine lower extremity neuromuscular function during gait in patients receiving either VR or TC treatment, and to examine how changes in neuromuscular patterns relate to changes in trunk control. Our first hypothesis, that patients receiving TC treatment will improve lower extremity motor control by increasing ankle MEE contribution and decreasing hip MEE contribution more so than the VR patients was not supported by analysis of between-group differences. However, in examining the within-groups changes (pre versus post-intervention), some potentially important biomechanical observations were made. Our second hypothesis, that trunk control in patients receiving TC will be positively correlated with improvements in lower extremity motor control, but trunk control in those receiving VR will not be positively correlated with improvements in lower extremity motor control, was supported.

Although an overall improvement in gait function (as indicated by time-distance measures) for both treatment groups was observed, confirming our general hypothesis, our data suggest that the mechanisms underlying those improvements differ, and appear to be linked to differences in neuromuscular responses of the lower extremities to the treatment programs. Specifically, our data suggest that changes in the relative contribution of individual joints to total leg mechanical energy expenditure (MEE), and the relationship between changes in lower extremity mechanical energy expenditure and changes in upper body kinematics, are different between TC and VR interventions (Figure 4). Further, these data highlight the importance of assessing gait not only with time-distance functional gait measures (Table 1), but also with measures that assess neuromuscular function of the lower extremities and control of the body's most massive segment, the trunk.

We found that TC patients significantly increased the contribution of ankle MEE to total leg MEE and decreased contribution of hip MEE to total leg MEE following the treatment program, while the VR group showed no significant change in ankle or hip MEE contributions following intervention (Figure 2). Although the changes were not statistically different between groups, the within groups comparisons suggest that clinically important trends may nonetheless be present in terms of biomechanical responses to the different therapies. Figure 1 shows the total joint MEE^(t) ^(sum of all transfer components) for ankle, knee and hip, and total leg MEE^(t)^, for each group. The increases in leg MEE^(t)^, which were similar for both groups, were apparently achieved by different neuromuscular adaptations of the individual joints. Where the VR group appear to increase total MEE^(t) ^of each joint to gain a total MEE increase of the leg, the TC group show a distinctive pattern of substituting ankle plantar-flexor contribution for hip extensor/flexor contribution. Prior studies on the relative roles of ankle and hip kinetics in gait [19,20,24] suggest that the result observed for TC patients indicates a trend toward a reduction in hip compensation and increased use of ankle muscles to provide both propulsion and stability.

Figure 2 shows the percent contribution of the changes to the concentric leg MEE^(+) ^for the changes in concentric MEE^(+) ^of individual joints. While both groups decreased the relative contribution of concentric hip MEE^(+)^, only the TC group increased the contribution of ankle concentric MEE^(+)^, while the VR group increased the contribution of knee concentric MEE^(+)^. Concentric energy transfer represents the energy expended by muscles in concentric contraction when energy is being transferred between segments. Because concentric contraction represents work being done *by *the muscles (as opposed to eccentric contraction, which is work being done *on *the muscles), we can interpret the above finding as meaning that, for the TC group, a greater proportion in the change in concentric work done by the leg muscles is attributed to the change in concentric work of ankle plantar-dorsiflexors, while for the VR group, this contribution decreases.

One possible reason concentric ankle MEE contribution may have increased significantly in the TC group, but not the VR group, is because the TC and the warm-up exercises improved ankle flexibility. Tight ankles (limited range of motion, ROM) may preclude the optimal structural alignment to coordinate mechanical energy sufficiently to increase propulsion [41], perhaps at the expense of trunk stabilization. Ankle function is important for balance corrections in both healthy elderly and vestibulopathic subjects [42-44]. A study by Van Deusen et al. [45] found that Tai Chi-like exercises for elders with arthritis resulted in a significant increase in ankle plantar flexion; this finding supports the above contention that the TC group in our study may have increased ankle MEE contribution as a result of increased ankle ROM, ankle moment, or both. The tight coupling between ankle and hip power in gait [19] would also explain the neuromuscular adaptive decrease in hip MEE contribution. Given the importance of ankle-plantar flexors in both propulsion and trunk stability, we conclude that TC teaches optimization of MEE in an effort to control the trunk while improving lower extremity function. The relationship between lower extremity MEE and trunk kinematics for the two treatment groups lends further credibility to this conclusion.

As shown in Figure 4, the relationship between change in leg MEE and change in the range of forward trunk velocity was positive for the TC group, and negative for the VR group. Similar relationships were also observed between change in leg MEE and change in peak forward trunk velocity. The observed direct relationship for the TC group suggests that the redistribution of power among ankle, knee and hip joints, which resulted in a net increase in the total MEE of the leg, enabled these patients to attain a faster gait. This observation is expected based on the principles of TC, which emphasize a vertical alignment integrating the head, torso, hips and legs. This concept of integrated alignment is reflected in phrases from the TC classics such as ". suspend the spine like a necklace of pearls" and "movements are initiated in the feet, steered by the waist and administered through the hands." [46]. In contrast, for the VR group, however, the increase in leg MEE was associated with a decrease in both peak and range of trunk velocity. This finding suggests that VR subjects, when increasing power generation/absorption with their lower extremities, reduce trunk oscillations during gait, possibly as a way to stabilize the trunk and head. This corrective procedure may not be necessary for TC subjects as they learned to move the trunk more proportionately to total lower extremity MEE, without need to explicitly attend to additional factors or mechanisms to stabilize the head. Although speculative, these scenarios correspond with the observed high positive correlation between change in leg MEE and change in trunk velocity peak and range seen after TC training but not after VR rehabilitation.

Because the VR exercise program may increase subjects' awareness of eye and head movement strategies that cause dizziness and instability [8,47], a more rigid head and trunk strategy during dynamic activities such as gait would be expected. The VR program's balance retraining exercises do not emphasize dynamic whole body movement patterns that improve overall postural control [10]. Subjects practice maintaining balance in challenging postures (narrow base of support such as feet together and one-legged standing still) using a variety of self selected movement patterns. It is probable that subjects would make the trunk more rigid to lessen head movement during these tasks. The VR group's decrease in trunk velocity range with increase in mechanical energy of the lower extremities during gait appears to support this explanation. Within the TC group, the subjects practice series of movement patterns that include elements of controlled trunk rotation without instruction on eye fixation. The training of smoothly transitioning body segment motions may provide these subjects a different mode of compensation for their instability. Practice of the TC movements may promote more natural trunk movements similar to healthy persons as shown in our biomechanical findings during gait.

Although the preliminary results presented here suggest the lower extremities may play an important role in the ability of vestibulopathic patients to improve gait function, several limitations of the present study may prevent broad generalization of the results. Our small sample size was perhaps the most important limitation. It is probable that the lack of between-group significant changes, particularly in light of many significant within group differences (pre-post intervention), was due to high variances obscuring group mean differences. Indeed, a larger sample size for controlling type II errors (increasing power), and better control of type I errors for multiple statistical tests, is warranted for future full-scale studies. The large age range within groups may have also contributed to high variability, but note that more heterogeneous samples in fact enhance external validity, including generalizability, of the results. We also did not include a no-treatment control group in the experimental design of this preliminary study. Because vestibulopathic patients may learn to compensate spontaneously, a no-treatment or sham-treatment group would be necessary to determine if changes in gait function are truly a result of the interventions. This is unlikely, however, given the inclusion criteria that all subjects must have had stable symptoms for 6 months and were on average 3 year post-onset of vestibulopathy. Given that both groups improved gait function in our randomized design comparing substantially different interventions suggests that the effects observed were not spurious. It must be recognized, however, that assumed improvements in function, via increased gait speed for example, may be limited [48,49]. As well, we only analyzed the mechanics of the lower extremities in the sagittal plane. It is highly likely that compensations for lower limb power impairments occurred in frontal and transverse planes as well. Also, the different number of patients in each group having a diagnosis of UVH and BVH is a potential limitation. Although there were no significant differences in proportion of UVH and BVH between the two treatment groups, that the BVH patients were much more disabled than the UVH patients in this study may be important, even when the difference in proportions of diagnostic categories (BVH or UVH) within treatment groups is small. Lastly, although there were no significant differences in age and gender distribution between treatment groups, a larger study sample would allow such subgroup effects to be studied.

We conclude that VR and TC can successfully improve gait function, as determined by common time-distance measures, in patients with vestibulopathy. We further conclude, however, that TC improves lower extremity motor control more than VR, by selective redistribution of joint energetics, which appears to engender a more vigorous gait and better trunk control. TC, as a complementary treatment to VR, may allow for better control of the trunk through reorganization of lower-extremity motor patterns, elicited from the flowing, controlled TC exercises.

## Competing interests

The author(s) declare that they have no competing interests.

## Authors' contributions

All authors participated in the overall study design, contributed to the interpretation of data and writing/editing of the manuscript, and have read and approved the final manuscript. CAM conceived the hypotheses for this manuscript and carried out the data analysis; DEK was the principal investigator of the project; SWP was the neurologist associated with the project; DMS conducted the patient testing and assisted in development of the vestibular rehabilitation program; PMW conducted the Tai Chi intervention; and SLW was the project consultant.

## Pre-publication history

The pre-publication history for this paper can be accessed here:


